# Ante‐mortem interventions for deceased donation: legal barriers and uncertainty in Australia's decision‐making frameworks

**DOI:** 10.5694/mja2.70020

**Published:** 2025-08-05

**Authors:** Shih‐Ning Then, Dominique E Martin, Helen I Opdam

**Affiliations:** ^1^ Australian Centre for Health Law Research Queensland University of Technology Brisbane QLD; ^2^ Deakin University Geelong VIC; ^3^ Austin Health Melbourne VIC; ^4^ Australian Organ and Tissue Authority Canberra ACT

**Keywords:** Transplantation, Legislation, medical

Definitive decision making about deceased donation of organs and tissues usually occurs towards the end of a person's life. If possible, pathways to organ donation will depend on the clinical circumstances: donation after neurological determination of death (“brain death”), or donation after circulatory determination of death (DCDD). DCDD typically applies when ceasing life‐sustaining interventions is planned, and when death is expected within a timeframe permitting recovery of organs for transplantation (ie, controlled DCDD).^1^ People undergoing voluntary assisted dying may also choose to pursue DCDD.^2^

While donation decision making is a distinct choice, it is entwined with other important decisions that may arise throughout the end‐of‐life care period, such as decisions to:

- stop life‐sustaining treatment, (ie, supportive treatment initiated with the goal of facilitating recovery);
- undergo interventions before or after death that increase the chance of successful transplantation; and
- participate in research relating to donation or transplantation.

Although decisions are often interconnected and discussed contemporaneously, legal frameworks underpinning decision making for these decisions have developed separately and are often ill‐suited to integrated decision making at the bedside.^3^ This presents a barrier to implementation of important strategies — proven or currently experimental — to expand the pool of potential donors, and to ensure that organs recovered are successfully transplanted.

Specific clinical interventions that are initiated before death (ie, ante‐mortem interventions [AMIs]) may help preserve opportunities for donation or improve the outcomes of transplantation. Interventions range from blood tests to invasive procedures, such as elective non‐therapeutic intubation and ventilation; and risks and burdens can vary substantially.^3^, ^4^ Complicating ethical decision making, across Australia there is uncertainty about the legality of, and consent requirements for, AMIs and no agreed definition.^5^ This uncertainty particularly affects health care workers caring for potential donors, and may discourage or delay time‐critical decisions about the use of AMIs.

Ethical and clinical guidance is currently limited. The Australian and New Zealand Intensive Care Society (ANZICS) statement on death and organ donation,^6^ for example, notes that where the use of AMIs is lawful, consent should be obtained from the individual or their family. It does not clarify how decisions should be made or who may have legal authority for decision making. Consequently, uncertainty and disagreements are common at the local level. Hospital staff and executives, together with donation agency staff may face questions about cases such as Arwen's (Box 1), where substitute decision making about use of AMIs is required.

Box 1 **Hypothetical case study**

**Table:** 

Arwen is a 45‐year‐old man who has had a catastrophic brain injury in a scooter accident and lives in an Australian capital city. Despite timely neurosurgery, there is no hope of Arwen recovering meaningful brain function; however, he does not meet the diagnostic neurological criteria for death. Arwen's wife (Sascha), following discussions with the intensive care unit (ICU) and trauma team, understands the need to cease life‐sustaining treatment and transition to comfort care. Arwen has been unconscious for six days and is expected to remain so until his death, which is likely to occur soon after removal of assisted ventilation and circulatory support. Sascha informs the ICU staff that Arwen is a registered organ donor, and that they had several conversations about their mutual goal to donate organs if the opportunity arose. The donation coordinator is contacted and meets with Sascha to explain the donation after circulatory determination of death pathway and obtain consent for donation. Sacha, a dialysis nurse, has read about ante‐mortem interventions (AMIs) and asks if any intervention can be used to give Arwen the best chance of successfully donating his organs for transplantation.
Consider, for example, if the clinicians could lawfully perform a bronchoscopy in Arwen to assess and increase the possibility of recovery and transplantation of lungs? If they can, who should they ask to provide lawful consent?
This article shows that, legally, the answer will differ depending on:
where Arwen lives in Australia; and
if the AMI is part of an approved medical research study.
In some Australian jurisdictions, the answer to who can provide consent to AMIs is very unclear.
In jurisdictions such as Victoria and New South Wales, where specific laws provide a pathway for consent to AMIs, Sascha can consent on behalf of Arwen to AMI procedures including the administration of medication, medical imaging and removal of blood for testing. Sascha is also the person who can legally authorise Arwen to become a donor upon his death.
In other jurisdictions, while Sascha will still be the person who can legally authorise Arwen to be a deceased donor following death, there will be less certainty that she can consent to AMIs before Arwen's death. Clinical guidelines note the absence of legal certainty on this issue,^5^ which may lead clinicians to act more conservatively or not seek consent for some types of AMIs. Consequently, some opportunities for donation may be missed if AMIs that would increase the probability of successful recovery and transplantation of organs are not agreed upon and implemented in a timely manner.

## Complexity in legal frameworks governing decision making from life to death

In Australia, medical decision making for adults lacking decision‐making capacity is governed by legislation (ie, guardianship, substitute decision‐making, or medical decision‐making legislation). However, at the end of life, when deceased donation may be considered, other legal frameworks may become relevant, and potential mismatches between frameworks within states and territories and across Australia become problematic. Box 2 shows statutory frameworks relevant to substitute decision making during the end‐of‐life period and donation after death (ie, medical treatment, research, AMIs and donation).

Reliance on legal frameworks that use the declaration of death as a demarcation is also complicated because what appears to be a clear legal dividing line is not easily navigated in many clinical situations (Box 3, A and B). For example, substitute decision makers for treatment may differ from people tasked with the donation decisions that take effect following death.^7^

Box 2 **Designated substitute decision maker and legislation across Australia for medical, research and deceased donation related decisions**

**Table:** 

State/territory	Medical treatment including end‐of‐life care*	Ante‐mortem interventions	Research participation (while individual is alive)*	Deceased donation
Australian Capital Territory	**Guardian, health attorney, attorney** (under an enduring power of attorney), (*Guardianship and Management of Property Act 1991*, *Powers of Attorney Act 2006*)	**Unclear**	**Guardian, medical research power of attorney** — for medical research or low risk research (*Guardianship and Management of Property Act 1991*, *Powers of Attorney Act 2006*)	**Senior available next of kin** (*Transplantation and Anatomy Act 1978*)
Northern Territory	**Health care decision maker** (can include guardian and person appointed under advance personal plan), (*Health Care Decision Making Act 2023*)	**Unclear**	**Health care decision maker** — for research or a clinical trial; **tribunal** — for special medical research or experimental health care, (*Health Care Decision Making Act 2023*)	**Senior available next of kin** (*Transplantation and Anatomy Act 1979*)
New South Wales	**Enduring guardian, person responsible** (can include guardian), (*Guardianship Act 1987*)	**Senior available next of kin** (*Human Tissue Act 1983*)	**Tribunal or person responsible** (where specific authority is conferred) — for approved clinical trials and special treatment (including new treatments), (*Guardianship Act 1987*)	**Senior available next of kin** (*Human Tissue Act 1983*)
Queensland	**Guardian, enduring power of attorney, statutory health attorney** (*Guardianship and Administration Act 2000*, *Powers of Attorney Act 1998*)	**Unclear**	**Tribunal** — for special medical research or experimental health care; **guardian, enduring power of attorney, statutory health attorney** — for approved clinical research, (*Guardianship and Administration Act 2000*, *Powers of Attorney Act 1998*)	**Senior available next of kin** (*Transplantation and Anatomy Act 1979*)
South Australia	**Person responsible** (can include guardian), **substitute decision‐maker**, (*Advance Care Directives Act 2013*, *Guardianship and Administration Act 1993*, *Consent to Medical Treatment and Palliative Care Act 1995*)	**Unclear**	Not explicitly provided for in legislation	**Senior available next of kin** (*Transplantation and Anatomy Act 1983*)
Tasmania	**Person responsible** (can include guardian) (*Guardianship and Administration Act 1995*)	**Unclear**	**Person responsible** for health and medical research (*Guardianship and Administration Act 1995*)	**Senior available next of kin** (*Human Tissue Act 1985*)
Victoria	**Medical treatment decision maker** (can include guardian), (*Medical Treatment Planning and Decisions Act 2016*, *Guardianship and Administration Act 2019*)	**Medical treatment decision maker** (*Human Tissue Act 1982*)	**Medical treatment decision maker** for a medical research procedure (*Medical Treatment Planning and Decisions Act 2016*)	**Senior available next of kin** (*Human Tissue Act 1982*)
Western Australia	**Enduring guardian, guardian, person responsible** (*Guardianship and Administration Act 1990*)	**Unclear**	**Research decision maker** for medical research (*Guardianship and Administration Act 1990*)	**Senior available next of kin** (*Human Tissue and Transplant Act 1982*)

* The information in these columns assumes that relevant substitute decision makers have been granted powers in relation to treatment or research. The statutory definitions and scope of these differ between jurisdictions’ legislation and will depend on the type of treatment or research proposed.

Box 3 **Comparison of how legal frameworks and clinical decision making differ across the end of life period in Australia**

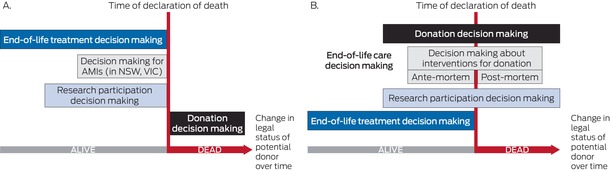

AMIs = ante‐mortem interventions; NSW = New South Wales; VIC = Victoria. * (**A**) Representation of how the law in Australia categorises different decisions, often with different considerations for designated substitute decision makers. (**B**) Representation of how clinical decision making may occur, indicating the substantial potential overlap of decision making during the end‐of‐life period, regardless of legal status of the potential donor

## Ante‐mortem interventions in legal decision‐making frameworks

AMIs are unusual medical interventions not aimed at improving a person's physical health, but at maximising the success of something after death. Therefore, AMIs do not fit neatly into decision‐making frameworks designed for choices affecting people during their lives. The ability of substitute decision makers to authorise or consent to AMIs is unclear in most Australian jurisdictions outside of Victoria and New South Wales, which have dedicated laws governing AMIs.^8^, ^9^

## The non‐therapeutic problem

There is uncertainty whether AMIs fall within the scope of decision‐making authority granted to substitute decision makers because of the way legislation defines terms such as “health care” or “medical treatment”. In some jurisdictions, the terms seem limited to interventions with therapeutic aims — at odds with some types of AMIs that offer no intrinsic therapeutic value to the prospective donor.

For example, the term “health care” in Queensland and South Australia is defined as meaning any care, service, procedure or treatment provided by, or under the supervision of, a health provider for the purpose of diagnosing, maintaining or treating a physical or mental condition of a person.^10^, ^11^, ^12^ While some forms of AMIs (eg, continuing mechanical ventilation or medications for blood pressure support) are arguably aimed at maintaining a person's physical condition, other AMIs are not.

In contrast, Western Australia, Tasmania and the Northern Territory have wider definitions that do not expressly include a therapeutic aim, instead referring broadly to treatment or health care that is part of a health service.^13^, ^14^, ^15^ There, AMIs administered by health professionals in hospitals are likely to fall within these wider definitions and thus the remit of substitute decision makers.

Lack of an agreed definition for AMIs adds to the uncertainty, leading to different practice across institutions and jurisdictions. For example, non‐invasive imaging such as computer tomography scanning has at times been refused, whereas more invasive investigations such as coronary angiography have sometimes been permitted. The recent legislative amendments in Victoria and NSW include definitions that provide greater clarity but specify different procedures as AMIs.^16^, ^17^

DCDD would be effectively impossible without certain non‐therapeutic procedures, such as obtaining blood samples for donor screening and matching. Legislation could explicitly list permissible AMIs or rely on regulations that can be updated more easily. However, it may be preferable simply to legally define AMIs as “any procedure undertaken before death for the purpose of organ donation that would not otherwise occur, including those to assess, maintain or improve the viability of organs for transplantation,” and leave decision making about their appropriateness as a matter for clinical and ethical guidance, rather than law, as is customary for most clinical procedures.

## Decision‐making principles

Uncertainty also relates to the various principles that substitute decision makers are required to consider in making decisions,^18^ especially those regarding how to recognise the (sometimes) competing interests of respecting a person's autonomy (their wishes) and promoting their health.

The significance of these principles was evident in NSW before legal changes. Before July 2024, the government considered that substitute decision makers could not consent to AMIs because they were required to ensure “that any medical or dental treatment … is carried out for the purpose of promoting and maintaining … [the person's] health and well‐being”.^19^, ^20^ The non‐therapeutic nature of AMIs meant they did not align with that goal and that no substitute decision makers could legally authorise AMIs. That position was reflected in the ANZICS *Statement on death and organ donation*.^6^ Similar language exists and can apply in the Australian Capital Territory to substitute decisions by enduring attorneys.^21^

In other jurisdictions, including Queensland, South Australia and the ACT (for substitute decision makers other than enduring attorneys) principles requiring consideration of an adult's wishes may be more prominent.^22^, ^23^, ^24^, ^25^, ^26^ Respect for autonomy, or the interest in governing one's own life and having control over one's body, requires consideration of the values, wishes and preferences of the person where these are known or may be estimated. Such principles may be articulated alongside others that require broader considerations in decision making which extend beyond the health of the individual, such as a person's best interests or promotion of their personal and social wellbeing (eg, Tasmania and Western Australia^27^, ^28^). Furthermore, contemporary substitute decision‐making principles, such as those recently enacted in the Northern Territory, establish that, in general, a person's wishes ought to be respected even if substitute decision makers believe these wishes are not in the best medical interests of the person, and if respecting their wishes will benefit another person (eg, through donation).^29^

## Ante‐mortem interventions in research

Uncertainty also applies to the use of AMIs in medical research.^30^ Provision of AMIs as part of research may provide an alternative to the usual medical decision‐making pathway, but additional legal conditions must normally be fulfilled. Once again, legal complications arise regarding who can consent to research participation (Box 2). This differs depending on whether the jurisdiction designates a specific research substitute decision maker (eg, ACT and WA)^31^, ^32^ and/or how the research intervention is classified (eg, low risk, experimental, clinical trial etc). Some states require additional legal authorisation, such as tribunal approval before substitute decision‐maker consent, for certain types of research (ie, clinical trials).^33^ These are in addition to the standard requirement for Human Research Ethics Committee approval.

In Victoria and NSW, where AMI‐specific provisions provide a pathway for decision making, AMIs offered in a research context may need additional legal requirements to be satisfied.^34^, ^35^ In some jurisdictions, the research pathway may offer a clearer legal avenue for administration of AMIs. Therefore, whether or not AMIs are administered in a research context may be significant.

## Conclusion

The legal complexities outlined here explain why substitute decision makers and clinicians may be uncertain about whether decisions on the use of AMIs are supported by law. Despite guidance in the *Ethical guidelines for cell, tissue and organ donation and transplantation* from the National Health and Medical Research Council,^36^ legal uncertainty constitutes a substantial barrier to efforts aimed at ensuring consistent good medical practice in end‐of‐life care and facilitating opportunities for donation and transplantation. With reforms in NSW and the Australian Law Reform Commission's review of human tissue legislation, reform is needed to provide a clearer path for legal authorisation for AMIs when these are deemed clinically and ethically appropriate.

## Open access

Open access publishing facilitated by Queensland University of Technology, as part of the Wiley – Queensland University of Technology agreement via the Council of Australian University Librarians.

## Competing interests

Shih‐Ning Then and Dominique Martin have acted as paid consultants to the Australian Organ and Tissue Authority in developing guidelines. Helen Opdam holds the role of National Medical Director of DonateLife (Organ and Tissue Authority).

## Provenance

Not commissioned; externally peer reviewed.

## Author contributions

Then SN: Conceptualization, writing – original draft and review and editing, formal analysis of the paper. Martin DE: Conceptualization, writing – review and editing. Opdam HI: Writing – review and editing.
